# Dual Allosteric Effect in Glycine/NMDA Receptor Antagonism: A Comparative QSAR Approach

**DOI:** 10.3390/ph3103167

**Published:** 2010-10-11

**Authors:** Manish Sharma, Vipin B. Gupta

**Affiliations:** 1 Centre for QSAR and Molecular Modeling, Department of Medicinal and Pharmaceutical Chemistry, TIFAC-CORE, Innovation Square, B. R. Nahata College of Pharmacy (Scientific and Industrial Research Organization), Mandsaur, 458001, MP, India; E-Mail: vbgupta@hotmail.com (V.B.G.); 2 Research Scholar at Jodhpur National University, Jodhpur (Raj), India

**Keywords:** allosterism, comparative QSAR, glycine/NMDA, CMR, quinoxalines

## Abstract

A comparative Hansch type QSAR study was conducted using multiple regression analysis on various sets of quinoxalines, quinoxalin-4-ones, quinazoline-2-carboxylates, 4-hydroxyquinolin-2(1*H*)-ones, 2-carboxytetrahydroquinolines, phenyl-hydroxy-quinolones, nitroquinolones and 4-substituted-3-phenylquinolin-2(1*H*)-ones as selective glycine/NMDA site antagonists. Ten statistically validated equations were developed, which indicated the importance of CMR, Verloop’s sterimol L1 and Clog*P* parameters in contributing towards biological activity. Interestingly, normal and inverse parabolic relationships were found with CMR in different series, indicating a dual allosteric binding mode in glycine/NMDA antagonism. Equations reveal an optimum CMR of 10 ± 10% is required for good potency of antagonists. Other equations indicate the presence of anionic functionality at 4-position of quinoline/quinolone ring system is not absolutely required for effective binding. The observations are laterally validated and in accordance with previous studies.

## 1. Introduction

The Central Nervous System (CNS) is regulated by excitatory and inhibitory amino acids known as neurotransmitters. A striking balance between the two maintains normal harmony of CNS physiology and overall well-being of an individual. A slight unbalance precipitates typical CNS disorders [1,2,3,4,5,6]. Receptors for excitatory neurotramsmitters are classified into metabotropic (G-protein coupled receptors) and ionotropic (ligand gated ion channels) types [7]. Ionotropic receptors comprises of kainate, (*S*)-2-amino-3-(3-hydroxy-5-methylisoxazol-4-yl) propionic acid (AMPA), and *N*-methyl-D-aspartic acid (NMDA) receptors. NMDA receptors are permeable to sodium, calcium, and potassium ions. Of paramount importance is permeability to Ca^2+^ ions, which confers on them an important role in synaptic plasticity under physiological condition and neuronal death under pathological conditions. Different binding sites on NMDA receptors allows a vast number of allosteric (allostery, a word of Greek origin meaning “another shape”) interactions, namely: glycine, polyamine, phencyclidine, zinc, magnesium, phosphorylation, as well as sites, which are prone to modulation by different pH or redox states [8,9,10,11,12,13]. Therapeutic benefit of strychnine insensitive glycine site is currently under investigation for different acute and chronic disorders, e.g. Alzheimer’s disease, Amytrophic Lateral Sclerosos, Parkinson’s disease, Huntington’s Chorea, anxiety disorders, depressions, epilepsy, stroke, trauma and chronic pain [14]. The positive aspects of choosing glycine/NMDA receptor site as drug target are lack of vacuolization, learning impairment, or psychotomimetic effects, for glycine antagonists compared to competitive antagonists at the NMDA receptor or channel blockers.

Crum-Brown and Fraser [15] first laid the foundation of QSAR by proposing the idea that biological response is a function of chemical structure. A set of extra-thermodynamically derived and computationally based descriptors is employed to correlate *in vitro* or *in vivo* biological activity of a set of molecules. Generally such descriptors are lipophilic, electronic, steric or topologic in nature. Correlating these parameters with biological activity of a set of molecules provides valuable insight into molecular mechanism of binding interaction. However, choice of parameters is very important, as their availability has become quite handy. Numerous irrelevant parameters and their values are made available at the press of a key, which could not provide any information as to how a molecule is interacting with receptor. A stroke of chance could result in a statistically significant equation with one or other parameter. Although quite important, statistics alone could not discount the doubt and risk involved in a standalone QSAR equation. In the words of Ernest Rutherford, “*If your experiment needs statistics*, *you ought to have done a better experiment.*” 

Comparative QSAR is the only methodology, which is ideally suitable to validate any given equation. It focuses upon the idea that same biological system (target receptor or enzyme) when interacts with different sets of series, the physicochemical properties involved in drug-receptor interaction generally remain similar [16]. Such equations laterally validate the observation obtained and attract authenticity. Comparison of such QSAR equations assists in understanding the mechanistic interpretation of drug-receptor binding and nature of subsite. Newer series could thus be proposed for further synthesis keeping the observations recorded from the equations and screening molecules through Lipinski’s “rule of five” for bioavailability check. Potential analogs from the proposed series could be further tested in tandem with molecular docking simulations to inspect whether they could potentially inhibit or stimulate the corresponding receptor subsite. 

In present study, we have developed ten QSAR equations for various sets of selective glycine/NMDA antagonists to understand the ligand-receptor interaction and laterally validate the observations.

## 2. Experimental Section

### 2.1. The Data Set

Multiple regression analysis was performed to carry out QSAR study with Hansch approach on glycine site inhibiting activity of NMDA receptor of ten different series of quinoxalines, quinoxalin-4-ones, quinazoline-2-carboxylates, 4-hydroxyquinolin-2(1*H*)-ones, 2-carboxytetrahydroquinolines, phenylhydroxyquinolones, nitroquinolones and 4-substituted-3-phenylquinolin-2(1*H*)-ones to produce 50% inhibition of [^3^H] glycine or [^3^H]-L-689,560 binding to rat brain cortical membranes. The biological activity IC_50_ of the compounds was collected from the literature and converted into molar concentrations. A negative logarithm of biological activity was used provide better correlations with parameters and avoid clustering of data points; –logIC_50_ therefore becomes dependent variable in subsequent equations. The data table depicts various biological activities viz. observed by experimentation (Obs.), calculated by equation (Cal.), and externally predicted (Ext. Pred.). Δ indicates difference between Obs. and Cal. or Ext. Pred. activities.

### 2.2. Parameter Calculation

Physicochemical parameters for substituents of unionized molecules like Clog*P*, CMR and Verloop’s sterimol [17] L1 was obtained from *MMP plus™* (www.norgwyn.com). Molar refractivity is based on Lorentz-Lorenz equation: MR = (*n*^2^ − 1)/(*n*^2^ + 2)(*MW/d*) where *n* is the index of refraction, *MW* is molecular weight of the compound and *d* is density. An interesting facet of MR is its dependency on combined effect of molecular volume and polarizability represented by *MW* and *n*, respectively. However the effect of polarizability is less as for most compounds the *n* is in the range of 1.35–1.60 [18]. It is assumed that MR is a much better parameter than molecular volume [19]. A strong correlation indicates absence of polarizability effects and thus only bulkiness of the drug might be important in interacting with the receptor. To bring MR with the level of π it was scaled down by 0.1. MR with a positive coefficient indicates involvement of dispersive forces in drug receptor interaction [20]. 

### 2.3. Chemometric Tools and Technique

A multiple linear regression based software *QSAR* (received from BITS-Pilani, India) generates QSAR equations and provides correlation coefficient (*r*), standard deviation (*s*), and ratio between variance of calculated and observed activites (*F*); *F* = f*r*^2^/[(1 − *r*^2^)m], where f is degree of freedom, m is number of variables, f = n − (m + 1), where n is number of data points. *F* value indicates true relationship or level of significance of QSAR equation.

In equations, the figure in parentheses is 95% confidence intervals and *F* value in parenthesis is critical 99% confidence intervals. The software also provides intercorrelation matrix between descriptors. For internal validation, *r*^2^ and *r*^2^_A_ were calculated to assess “goodness of fit” of the equation. *r*^2^ value is variance between observed and calculated biological activity. An adjusted *r*^2^ is *r*^2^_A_ and calculated by the formula, *r^2^_A_ = r^2^(1 − 1/F)*. *Q*^2^ is cross-validated leave-one-out (L-O-O) *r*^2^ between observed and predicted activity [21]. It measures quality of QSAR equation. Another statistical index to estimate the quality is *Q*_y_, which is calculated by the ratio *r*/*s*. A high *Q*_y_ (high *r* and low *s*) indicates less probability of chance correlation between biological activity and parameters [22]. A squared correlation was also calculated between CMR and Clog*P* in each equation to ascertain the true role of each parameter. 

For testing the validity of a model, 25% compounds of the training set were removed randomly to constitute a test set. A QSAR equation was generated on remaining compounds to predict activity of test set molecules. The activity in all series save carboxytetrahydroquinolines refers to molar concentration of the compound required to displace [^3^H]-L-689,560 binding to glycine site of NMDA receptor in rat brain membranes. In carboxytetrahydroquinolines, the displaced compound is [^3^H]Gly. Compounds were deemed to be outliers on the basis of their difference between observed and calculated activities, which should be greater than 2*s*. Biological activity of outliers was calculated from the final equation. Applicability domains of QSAR models were estimated wherever necessary by software *AMBIT* [23].

## 3. Results and Discussion

For all ten series of glycine site antagonists of NMDA receptors (**I–VIII**), as listed in Table 1, Table 2, Table 3, Table 4, Table 5, Table 6, Table 7, Table 8, the best correlations (Equations 1–10) obtained are summarized below along with externally validated Equations (1a–10a):

### i. Inhibition of glycine/NMDA site by I (Table 1)* [24]*

− logIC_50_ = 0.157(0.100)CMR + 1.139 (0.363)*I*_1_ + 4.000(0.852)

*n* = 13, *r*=0.950, *s*=0.18, *F*_2,10_ = 45.93(7.56)

*r^2^* = 0.90, *r^2^_A_* = 0.88, *Q^2^* = 0.83, *Q_y_* = 5.28 

CMR range = 6.21-10.16, outliers = 20, 22 

*r^2^*(CMR *vs.* Clog*P*) = 0.63                                                                                                  (1)

− logIC_50_ = 0.131(0.124)CMR + 1.119(0.394)*I*_1_ + 4.277(1.049)

*n* = 10, *r* = 0.960, *s* = 0.17, *F*_2,7_ = 41.07(9.55)

*r^2^* = 0.92, *r^2^_A_* = 0.90, *Q^2^* = 0.84, *Q_y_* = 5.75

Excluded compounds = 8,14,17                                                                                           (1a)

The activity in Equation (1) is largely governed by CMR, which reveals that there is an overall dispersion interaction of the molecule with the target receptor. Indicator variable *I*_1_ takes a value of 1 for compounds with conformational constraints in the form of triple bond and zero, if otherwise. A positive *I*_1 _coefficient indicates high electronic density moieties in the form of triple bond add to the potency of the molecule by strengthening dispersive forces involved in ligand-receptor interaction. Interestingly, CMR is a very good measure of dispersive force. A small correlation between CMR and Clog*P* presumably indicates that lipophilicity of molecules is also important but we could not obtain a good correlation with Clog*P*. Compounds 20 and 22 were not included in the equation as they exhibited inconsistent activities.

**Table 1:** A series of 4-hydroxyquinolin-2(1*H*)-ones, 3-esters I, with their structural parameters and binding affinities. 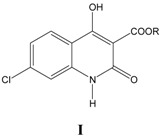

C.N.	R	*I*_1_	CMR	*−logIC50*				
				Obs.	Cal.	Δ	Ext. Pred.	Δ
8	Et	0.00	6.68	4.78	5.05	−0.27	5.15	−0.37
10	Me	0.00	6.21	5.19	4.98	0.21		
12	Allyl	0.00	7.09	5.19	5.12	0.07		
13	Propargyl	0.00	6.94	5.04	5.09	−0.05		
14	CH_2_-3-(phenol)	0.00	8.78	5.21	5.38	−0.17	5.43	−0.22
15	(CH_2_)_2_-3-(phenol)	0.00	9.24	5.74	5.45	0.29		
16	(CH_2_)_3_-3-(phenol)	0.00	9.70	5.58	5.52	0.06		
17	(CH_2_)_4_-3-(phenol)	0.00	10.16	5.58	5.60	−0.02	5.43	−0.03
18	(CH_2_)_2_-2-(phenol)	0.00	9.24	5.15	5.45	−0.15		
19	(CH_2_)_2_-4-(phenol)	0.00	9.24	5.53	5.45	0.08		
20	(CH_2_)_2_-2-(pyridine)	0.00	9.02	4.56^a^	5.42	−0.86		
21	(CH_2_)_2_-2-(thiophene)	0.00	9.01	5.52	5.42	0.10		
22	(CH_2_)_2_-3-(indole)	0.00	10.14	6.37^a^	5.59	0.78		
23	CH_2_C≡C-4-(anisole)	1.00	9.97	6.57	6.71	−0.14		
24	CH_2_C≡C-4-(phenol)	1.00	9.50	6.77	6.63	0.14		

^a^ outlier

### ii. Inhibition of glycine/NMDA site by II (Table 2)* [24]*

− logIC_50_ = − 0.440(0.219)Clog*P* + 0.230(0.070)Clog*P*^2^ + 4.771(0.161)

*n* = 5, *r* = 0.997, *s* = 0.04, *F*_1,3_ = 165.81(34.12)

*r^2^* = 0.99, *r^2^_A_* = 0.99, *Q^2^* = 0.83, *Q_y_* = 26.24 

Inversion point = 0.96(0.65 − 1.16)

*r^2^*(CMR *vs.* Clog*P*) = 0.032                                                                                           (2)

In the generation of Equation (2), activity seems to be dependent upon Clog*P* in an allosteric way. Activity decreases with Clog*P* upto inversion point and then increases. For good activity, Clog*P* of molecules atleast should be above inversion point (0.96). The observation gets support from the derivation of Equation (3), which indicates a positive linear relationship of biological activity with Clog*P* in arange above inversion point (1.22–2.70).

**Table 2:** A series of 3-(ethoxycarbonyl)-4-hydroxyquinolin-2(1*H*)-ones II, aromatic substitutions with their structural parameters and binding affinities. 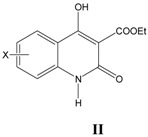

C.N	R	ClogP	*−logIC50*		
			Obs.	Cal.	Δ
29	7-CN	0.85	4.58	4.56	0.02
30	7-CF_3_	2.30	4.91	4.97	−0.06
31	7-NO_2_	1.16	4.60	4.57	0.03
32	5-I, 7-Cl	3.25	5.79	5.76	0.03
33	6,7-(NO_2_)_2_	−0.42	4.98	4.99	−0.01

### iii. Inhibition of glycine/NMDA site by III (Table 3)* [24]*

*−logIC50* = 0.601(0.369)Clog*P* + 4.448(0.788)

*n* = 5, *r* = 0.948, *s* = 0.11, *F*_1,3_ = 26.85(34.12)

*r ^2^* = 0.99, *r^2^_A_* = 0.99, *Q^2^* = 0.80, *Q_y_* = 8.62

outliers: 9, 42 *r*^2^(CMR *vs.* Clog*P*) = 0.352                                                                     (3)

**Table 3:** A series of 4-hydroxyquinolin-2(1*H*)-ones, 3-ketones III, with their structural parameters and binding affinities. 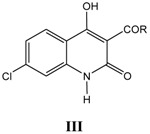

C.N.	R	Clog*P*	*−logIC50*		
			Obs.	Cal.	Δ
9	C_6_H_5_	2.70	5.50^a^	6.07	−0.57
35	3-thiophene	2.34	5.70	5.86	−0.16
36	3-furan	1.90	5.62	5.91	−0.29
37	3-pyridine	1.22	5.16	5.18	−0.02
39	CH_2_C_6_H_5_	2.64	6.03	6.03	0.00
40	CH_2_-3-(thiophene)	2.28	5.97	5.82	0.15
42	cyclopropane	1.51	6.38^a^	5.36	1.02

^a^ Outlier

### iv. Inhibition of glycine/NMDA site by IV (Table 4)* [25]*

*− logIC50* = − 0.235(0.076)L1 + 3.853(0.909)CMR

− 0.176(0.045)CMR^2^ − 0.549(0.260)*I*_2_ – 12.124(4.378)

*n* = 18, *r* = 0.960, *s* = 0.14, *F*_3,14_ = 38.44(5.56)

*r^2^* = 0.92, *r^2^_A_* = 0.90, *Q^2^* = 0.85, *Q_y_* =7 .06

CMR_o_ = 10.9(10.7-11.3), outlier = 22

*r^2^*(CMR *vs.* Clog*P*) = 0.37                                                                                                   (4)

*− logIC50* = − 0.239(0.121)L1 + 3.895(0.719)CMR

− 0.179(0.035)CMR*^2^* − 0.524(0.175)*I*_2_ – 12.311(3.306)

*n* = 12, *r* = 0.98, *s* = 0.07, *F*_3,8_ = 81.95(7.59)

*r^2^*= 0.98, *r^2^_A_*= 0.97, *Q^2^*= 0.94, *Q_y_*= 13.36

CMR_o_ = 11.00(10.80−11.30)

Excluded compounds = 21, 14, 29, 30, 31                                                                           (4a)

In Equation (4), again CMR emerges as an important parameter with normal allosteric effect. The CMR_o_ obtained is 10.9 (10.7-11.3). This equation is particularly interesting as it provides clues for further synthesis of promising molecules with an optimum CMR range. The negative coefficient of L1 indicates that probably a size limited lipophilic pocket is present near to substituent R in receptor, which does not allow lengthy substituents for efficient binding. Indicator variable *I*_2_ stands with a value of unity for compounds with saturated ring as substituent at R and zero, if otherwise. A negative *I*_2 _in equation reveals that saturated rings are not preferred probably because of their thin delocalized electron cloud, which renders them weak to participate in dispersive force interaction between molecule and receptor for binding. 

**Table 4:** A series of trans-4-amido-2-carboxytetrahydroquinolines IV, with their structural parameters and binding affinities. 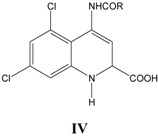

C.N.	R	CMR	L1	I2	*−logIC50*				
					Obs.	Cal.	Δ	Ext. Pred.	Δ
17	CH_3_	7.41	3.08	0.00	6.04	6.03	0.01		
18	*n*-Pr	8.33	5.28	0.00	6.43	6.50	−0.07		
19	c-hexyl	9.50	5.23	1.00	6.77	6.80	−0.03		
20	CH_2_-c-hexyl	9.96	5.50	1.00	6.96	6.93	0.03		
9	Ph	9.36	6.27	0.00	7.00	7.03	−0.03		
21	2-furyl	8.65	5.26	0.00	6.82	6.79	0.03	6.74	0.08
22	4-pyridyl	9.29	5.19	0.00	6.20^a^	7.26	−1.06		
14	CH_2_Ph	9.82	4.59	0.00	7.92	7.64	0.28	7.58	0.34
23	CH_2_(3-thienyl)	9.74	4.95	0.00	7.52	7.53	−0.01		
24	CH_2_(2-thienyl)	9.74	4.56	0.00	7.52	7.62	−0.10		
25	CH_2_C_6_H_4_NH_2_-4	10.16	5.28	0.00	7.70	7.60	0.10		
26	CH_2_C_6_H_4_OH-4	9.97	5.22	0.00	7.52	7.55	−0.03		
27	CH_2_C_6_H_4_CH_3_-4	10.28	6.00	0.00	7.52	7.46	0.06		
28	CH_2_C_6_H_4_OCH_3_-4	10.45	5.63	0.00	7.40	7.58	−0.18		
29	CH_2_C_6_H_4_Cl-4	10.31	5.62	0.00	7.30	7.55	−0.25	7.48	−0.18
30	(CH_2_)_2_Ph	10.28	8.59	0.00	7.10	6.85	0.25	6.76	0.34
31	(CH_2_)_3_Ph	10.74	8.54	0.00	6.77	6.93	−0.16	6.83	−0.06
32	CHPh_2_	12.23	5.80	0.00	7.22	7.28	−0.06		
33	9-fluorenyl	12.01	4.94	0.00	7.70	7.58	0.12		

^a^ Outlier

### v. Inhibition of glycine/NMDA site by V (Table 5)* [25]*

*− logIC50* = 0.430(0.193)CMR + 2.499(1.874)

*n* = 6, *r* = 0.952, *s* = 0.11, *F*_1,4_ = 38.40(21.20) 

*r^2^* = 0.90, *r^2^_A_* = 0.88, *Q^2^* = 0.67, *Q_y_* = 8.42

CMR range = 8.14-10.49, outlier = 57

*r^2^*(CMR *vs.* Clog*P*) = 0.31                                                                                               (5)

*− logIC50* = 0.412(0.222)CMR + 2.649(2.142)

*n* = 5, *r* = 0.960, *s* = 0.11, *F*_1,3_ = 34.91(34.12)

*r^2^* = 0.92, *r^2^_A_* = 0.89, *Q^2^* = 0.68, *Q_y_* = 8.97

Excluded compound = 54                                                                                               (5a)

Similarly CMR dominates Equation (5) with a positive regression coefficient in the range 8.14-10.49, which is a subset of CMR range witnessed in Equation (1), indicating both series **I** and **V** bind to the glycine site in a similar way. 

**Table 5:** A series of conformationally restricted 4-substituents **V**, with their structural parameters and binding affinities. 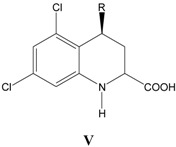

C.N.	R	*CMR*	*−logIC50*				
			Obs.	Cal.	Δ	Ext. Pred.	Δ
51	1-pyrrolidin-2-one	8.14	6.04	6.00	0.04		
52	1-isoindolin-1-one	9.63	6.43	6.64	−0.21		
53	2-isoindoline-1,3-dione	9.80	6.77	6.72	0.05		
54	2-(1,2-dihydroiso-quinolin-3-(4*H*) one)	10.09	6.96	6.84	0.12	6.81	0.15
55	1-(3-phenylimidazolidin-2-one)	10.49	7.00	7.01	−0.01		
56	3-(3,4-dihydroquinazolin-2-(1*H*)-one)	10.02	6.82	6.81	0.01		
57	-*N*-indoline-1-carboxamide	10.45	6.20a	6.99	−0.79		

^a^ Outlier

### vi. Inhibition of glycine/NMDA site by VI (Table 6)* [26]*

*− logIC50* = −13.60 (9.66)CMR + 0.633 (0.45)CMR^2^ + 81.45 (51.4)

*n* = 6, *r* = 0.940, *s* = 0.08, *F*_1,4_ = 11.42(21.20) 

*r^2^* = 0.91, *r^2^_A_* = 0.89, *Q^2^* = 0.64, *Q_y_* = 11.190

CMR range = 7.37−11.49, outlier = 6

Inversion point = 10.8(10.6−11.1), *r^2^*(CMR *vs.* Clog*P*) = 0.61 (6)

*− logIC50* = −14.61 (10.6)CMR + 0.681(0.50)CMR^2^ + 86.71 (56.5) 

*n* = 5, *r* = 0.975, *s* = 0.07, *F*_1,3_ = 19.49(34.12) 

*r^2^* = 0.95, *r^2^_A_* = 0.93, *Q^2^* = 0.80, *Q_y_* = 14.55

Excluded compound = 12 (6a)

In Equation (6), a negative allosteric effect with CMR is observed. Activity decreases with CMR upto inversion point (10.8) and then increases with further increase in CMR.

**Table 6:** A series of substituted 3-phenyl-4-hydroxy-2-quinolones VI, with their structural parameters and binding affinities. 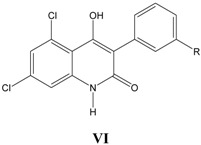

C.N.	R	*CMR*	*−logIC50*				
			Obs.	Cal.	Δ	Ext. Pred.	Δ
6	H	7.37	6.77a	15.56	−8.83		
9	CH_2_Ph	10.24	8.39	8.49	−0.10		
10	CH_2_PhOMe-4	10.87	8.35	8.33	0.02		
11	CH_2_PhOCH_2_OMe-4	11.49	8.66	8.67	−0.01		
12	CH_2_(3-thienyl)	10.16	8.64	8.54	0.10	8.57	0.07
13	OPh	9.94	8.70	8.74	−0.04		
14	O(3-thienyl)	9.86	8.85	8.83	0.02		

^a^ Outlier

### vii. Inhibition of glycine/NMDA site by VII (Table 7) *[27]*

*− logIC50* = −2.748(1.769)CMR + 0.140(0.093)CMR^2^

+ 1.660(0.766)*I*_a_ + 16.958(7.869) 

*n* = 10, *r* = 0.918, *s* = 0.21, *F*_2,8_ = 10.67(8.65)

*r^2^* = 0.87, *r^2^_A_* = 0.83, *Q^2^* = 0.42, *Q_y_* = 4.41

Inversion point = 9.82(9.38-10.9), CMR range = 7.05-11.90

*r^2^*(CMR *vs.* Clog*P*) = 0.04 (7)

*− logIC50* = −3.215(1.984)CMR + 0.162(0.105)CMR^2^

+ 1.792(0.793)*I*a + 19.166(8.815)

*n* = 7, *r* = 0.976, *s* = 0.14, *F*_2,5_ = 19.73(13.27)

*r^2^* = 0.95, *r^2^_A_* = 0.92, *Q^2^* = 0.77, *Q_y_* = 7.18 

Inversion point = 9.93(9.48-11.1) 

Excluded compounds = 4, 16, 19 (7a)

Equation (7) involves inverse allosteric effect with an inversion point of 9.82. Indicator variable *I*_a_= 1 for compounds containing acidic moieties and zero for neutral and basic moieties. A positive *I*_a_ indicates presence of acidic substituents is preferred which interacts with a proton acceptor site present in the glycine site close to R.

**Table 7:** A series of 3-nitro-3,4-dihydro-2(1*H*)-quinolones **VII** with their structural parameters and binding affinities. 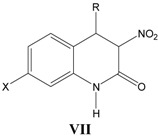

C.N.	R	X	*I*_a_	CMR	*−logIC50*				
					Obs.	Cal.	Δ	Ext. Pred.	Δ
4	OH	Cl	1.00	7.53	5.88	5.86	0.02	5.85	0.03
13	OCH_2_COOMe	H	0.00	7.05	4.54	4.54	0.00		
14	OCH_2_COOH	Cl	1.00	7.51	5.65	5.88	−0.23		
15	OCH_2_CONMe_2_	Cl	1.00	9.56	5.25	5.14	0.11		
16	OCH_2_CONH_2_	Cl	1.00	11.44	5.74	5.50	0.24	5.39	0.35
17	OCH_2_CN	Cl	1.00	10.94	5.12	5.30	−0.18		
18	OCH_2_-Pyr-2	Cl	1.00	9.00	4.91	5.22	−0.31		
19	O(CH_2_)_2_NMe_2_	Cl	1.00	9.49	5.43	5.14	0.33	5.02	0.41
20	OCH_2_COMe	Cl	1.00	11.90	5.60	5.74	−0.14		
21	OCH_2_C(Me)=NOH	Cl	1.00	7.06	6.38	6.19	0.19		

### viii. Inhibition of glycine/NMDA site by VIII (Table 8) *[28]*

*− logIC50*= 7.965(3.677)CMR − 0.397(0.180)CMR^2^

+ 1.162(0.319)*I*a − 34.426(18.651)

*n* = 9, *r* = 0.977, *s* = 0.13, *F*_2,6_ = 34.32(10.92)

*r^2^* = 0.95, *r^2^_A_* = 0.93, *Q^2^* = 0.84, *Q_y_* = 7.29

CMR_o_ = 10.0(9.76-10.20) CMR range = 7.56-11.56 

Outliers = 3,16, *r^2^*(CMR *vs.* Clog*P*) = 0.10 (8)

*− logIC50* = 9.201(5.698)CMR − 0.457(0.280)CMR^2^

+ 1.217(0.517)*I*a − 40.614(28.777)

*n* = 7, *r* = 0.977, *s* = 0.12, *F*_2,4_ = 20.85(18.00)

*r^2^* = 0.96, *r^2^_A_* = 0.94, *Q^2^* = 0.57, *Q_y_* = 7.88

CMR_o_ = 10.1(9.69−10.3), Excluded compounds = 6, 12 (8a)

In derivation of Equation (8), where heteroatom N of substituent R_1_ is connected to 3-phenylquinolin-2(1*H*)-one nucleus, a positive allosteric effect is observed. Activity increases upto inversion point (CMR_o_ = 10) and then starts decreasing. Preference for acidic substituents is indicated by a positive coefficient of *I*_a_. This equation could provide some clue in proposing molecules falling in optimum CMR range (9.76-10.20).

**Table 8:** A series of 4-substituted-3-phenylquinolin-2(1*H*)-ones **VIII** with their structural parameters and binding affinities. 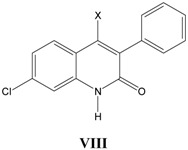

C.N.	X = R_1_	*I*a	CMR	*−logIC50*				
				Obs.	Cal.	Δ	Ext. Pred.	Δ
3	NH_2_	0.00	7.56	5.17^a^	3.10	2.07		
4	NHCOMe	0.00	8.67	4.83	4.81	0.02		
6	NHSO_2_Me	1.00	9.62	6.57	6.65	−0.08	6.82	−0.25
7	NHSO_2_Ph	1.00	11.57	5.92	5.79	0.13		
10	NHCH_2_Ph	0.00	10.45	5.70	5.49	0.21		
11	NH(CH_2_)_2_NMe_2_	0.00	9.82	5.49	5.54	−0.05		
12	NH(CH_2_)_3_NMe_2_	0.00	10.28	5.38	5.53	−0.15	5.68	−0.30
13	NHCOCOOH	1.00	9.00	6.24	6.29	−0.05		
14	NHCOCOOMe	0.00	9.47	5.57	5.43	0.14		
15	NHCOCONH(CH_2_)_2_NMe_2_	0.00	11.45	4.60	4.77	−0.17		
16	NHCOCH_2_COOH	1.00	9.46	5.96^a^	6.56	−0.60		

^a^ Outlier

### ix. Inhibition of glycine/NMDA site by VIII (Table 9) *[28]*

*− logIC50* = 0.647(0.313)*I*a − 1.289(0.325)CMR + 17.447(2.970)

*n* = 9, *r* = 0.974, *s* = 0.16, *F*_2,6_ = 56.05(10.92) 

*r^2^* = 0.95, *r^2^_A_* = 0.932, *Q^2^* = 0.91, *Q_y_* = 6.24 

outlier = 1, CMR range = 4.94-6.96

*r^2^*(CMR *vs.* Clog*P*):0.03 (9)

*−logIC50* = 0.687(0.279)*I*a − 1.237(0.285)CMR + 16.928(2.641)

*n* = 7, *r* = 0.990, *s* = 0.11, *F*_2,4_ = 99.79(18.00)

*r^2^* = 0.98, *r^2^_A_* = 0.97, *Q^2^* = 0.94, *Q_y_* = 9.25 

Excluded compounds = 22,27 (9a)

In Equation (9), where heteroatom O of substituent R_2_ is connected to 3-phenylquinolin-2(1*H*)-one nucleus, a negative linear relationship is observed with CMR. The equation also indicates a positive correlation with *I*_a_.

**Table 9:** A series of 4-substituted-3-phenylquinolin-2(1*H*)-ones **VIII** with their structural parameters and binding affinities.

C.N.	X = R_2_	*I*a	CMR	*−logIC50*					
				Obs.		Cal.	Δ	Ext. Pred.	Δ
1	OH	1.00	7.37	6.77^a^	7.30		−0.53		
19	OCH_2_COOMe	0.00	9.10	5.49	5.77		−0.28		
20	OCH_2_COOH	1.00	8.63	6.96	6.17		0.79		
21	OCH_2_CONMe_2_	0.00	9.80	4.94	5.18		−0.24		
22	OCH_2_CONH_2_	0.00	8.82	6.38	6.03		0.03	6.01	0.37
23	OCH_2_CN	0.00	8.52	6.49	6.46		0.03		
25	OCH_2_-Pyr-2	1.00	9.77	5.55	5.60		−0.05		
26	O(CH_2_)_2_NMe_2_	0.00	9.62	4.96	5.00		−0.04		
27	OCH_2_COMe	0.00	8.94	5.77	5.87		−0.10	5.87	−0.10
28	OCH_2_C(Me)=NOH	1.00	9.26	6.11	5.61		0.50		

^a^ Outlier

### x. Inhibition of glycine/NMDA site by VIII (Table 10) *[28]*

*− logIC50* = 0.781(0.383)*I*a + 12.130(7.142)CMR 

− 0.662(0.389)CMR^2^ − 49.338(32.694)

*n* = 8, *r* = 0.959, *s* = 0.13, *F*_2,5_ = 15.37(13.27)

*r^2^* = 0.93, *r^2^_A_* =0.90, *Q^2^* = 0.77, *Q_y_* = 8.70

CMR_o_ = 9.15(8.89-9.40), CMR range = 8.00-10.16, outlier = 39

*r^2^*(CMR *vs.* Clog*P*) = 0.04 (10)

*−logIC50* = 0.830(0.422)*I*a + 11.264(7.739)CMR 

− 0.616(0.422)CMR^2^ − 45.436(35.382)

*n* = 7, *r* = 0.974, *s* = 0.11, *F*_2,4_= 18.53(18.00)

*r^2^* = 0.95, *r^2^_A_* = 0.92, *Q^2^* = 0.66, *Q_y_* = 8.70 

CMR_o_ = 9.14(8.83-9.43), Excluded compound = 4 (10a)

A normal allosteric effect is again seen in Equation (10) with CMR, where carboxylic acid derivatives are substituted at 4-position in **VIII**. Activity increases upto inversion point (CMR_o_=9.15) and then decreases with further increase in CMR. This equation again could provide clues in proposing newer molecules falling in the optimum CMR range. Acidic substituents are preferred as indicated by positive coefficient of *I*_a._

**Table 10:** A series of 4-substituted-3-phenylquinolin-2(1*H*)-ones **VIII** with their structural parameters and binding affinities.

C.N.	X=R	*I*a	CMR	*−logIC50*					
				Obs.	Cal.	Δ	Ext. Pred.	Δ
31	COOH	1.00	8.00	6.04	6.06	−0.02		
33	CH_2_COOMe	0.00	8.94	5.96	6.12	−0.16		
34	CH_2_COOH	1.00	8.46	6.80	6.62	0.18		
36	CH_2_CH_2_COOCH_3_	0.00	9.40	6.30	6.10	0.20	6.02	0.28
37	CH_2_CH_2_COOH	1.00	8.93	6.77	6.90	−0.13		
38	(CH_2_)_2_-5-(3-methyl-1,2,4-oxadiazole)	0.00	10.16	5.46	5.47	−0.01		
39	CH_2_CH_2_CONH_2_	0.00	9.12	6.72^a^	6.23	0.49		
40	CH_2_CH_2_CN	0.00	8.82	6.12	6.08	0.04		
41	(CH_2_)_2_-5-(1*H*-tetrazole)	1.00	9.86	6.62	6.59	0.03		

^a^ Outlier

A comparison of these ten equations elicits some very interesting points about the nature of the glycine site and the rersulting mechanistic interpretation of its binding interactions. Out of ten equations CMR was found to be important in eight. All the equations satisfy statistical requirements. In data tables, Δ values in two columns are in close proximity indicating strong correlation between internal and external predictivities.

Two different types of allosterism were observed in these equations: normal and inverse, especially with parameter CMR. To interpret contribution of CMR in equations is quite challenging. This parameter includes not only volume but also polarizability of molecule. Polarizability, in turn, is directional and attractive dispersive forces, arising out of the charge transfer reaction between molecule and receptor, cannot be established until both are of opposite nature.

In normal allosterism, activity increases in a linear fashion with CMR up to the inversion point where the quadratic term takes over and it decreases with further increase in CMR, resulting in a closed parabolic graph. Perhaps bulky molecules with some polarizability interact at an opportune position in receptor site and therefore activity increases. With further increase in bulkiness beyond the inversion point, steric hinderance of molecules affect binding and activity starts decreasing. Looking at Equations (4,8,10) with normal allosteric effects, the coefficient of linear portion of CMR in Equation (10) is thrice than in Equation (4) and twice than in Equation (8), indicating contribution of CMR in activity variation is more in Equation (10). A similar CMR_o_ of 10 (± 10%) and *s* (0.136, 0.134 and 0.130) in all three equations indicate similar binding mechanism. Lateral validation or comparison of QSARs thus establishes the authenticity of these three equations. In Equations (1,5), a positive linear relationship with CMR in the range 6.21-10.16 and 8.14-10.49, respectively, might show but not necessarily, normal allosterism. This could be revealed if further derivatives in higher CMR range are synthesized and tested. In Equations (2,3) although CMR is not present, still a weakly positive relationship similar to linear portion of Equation (4,8,10) is exhibite, suggestive of normal allosterism.

Interestingly, according to a belief, molecules with lower CMR than 10 (° 10%) not being active could be related to evolution in the receptor structure. Logistic modifications in structure and conformaiton of proteins made them insensitive towards unnecessary stimulation from various endogenous small ligands, which started appearing in cell cytoplasm and nucleoplasm with eons of time [18].

In inverse allosterism, activity decreases initially up to an inversion point and then increases with further increase in CMR resulting in an open parabolic graph. A negative allosteric effect is seen in Equations (6) and (7) with different coefficients of the linear portion of CMR. The CMR ranges where these equations hold applicable are similar [7.37–11.49 for Equation (6) and 7.05–11.90 for Equation (7)]. The point of inversion is 10.8 for Equation (6) and 9.82 for Equation (7). These two equations support each other’s observation; although, the variation in activity with CMR is different in each equation.

In Equation (9), a negative linear relationship with CMR in the range 4.94-6.96 could show inverse allosterism if some more derivatives are synthesized and tested in higher CMR range.

A bilinear relationship of Kubinyi type was also tested by the *Bilin* (www.kubinyi.de) software for equations where parabolic relationship was found, however the relationship was statistically insignificant.

Maximum effect of *I*_a_ is seen in Equations (7) and (8) with a large coefficient of 1.660 and 1.162 respectively. Since *I*_a _is present in Equations (7,8,9 and 10), where both allosteric types are exhibited, it is tempting to speculate, that these binding mechanisms do not depend upon presence of anionic functionalities or acidic moieties or any other high electronic density fragments as substituents in a glycine site antagonist. This speculation is supported by a study of Carling *et al.* [28], who inferred that anionic functionality is not absolutely required for good activity and glycine site antagonists with neutral and basic moieties at 4-position of 3-phenylquinolin-2(1*H*)-ones performed equally well.

Our results of dual allosteric modes of binding for series **VII** and **IV** and **V** gets support from another observation of Carling *et al.* [27] who studied difference in structure activity relationships of these three series and indicated that they do not bind in an identical manner.

Certain QSAR studies although not conducted exhaustively are noteworthy. McQuaid *et al.* [29] conducted a QSAR analysis on a few 3-phenyl substituted-4-hydroxyquinolin-2(1*H*)-one compounds as glycine site antagonists (Equation 11) and observed a negative linear relationship of Hammett constant at para position σ_p_ with binding affinity. This indicates a positive effect of electron donating substituents at phenyl ring towards potency. A high electronic density in phenyl ring is therefore preferred. 

− log*K*_i_ = 0.95(0.18) – 1.26(0.41)σ_p_

*n* = 6, *r*=0.84, *F* = 9.43, *p* = 0.04                                                                                       (11)

Fabio *et al.* [30] synthesized some 3-substituted indole-2-carboxylates and conducted a QSAR study on them. The Equation (12) obtained reveals a negative linear relationship of summed MR and π values of substituents present at *o*,*m* and *p* positions of 3-phenyl ring. A negative linear correlation was also obtained with σ_p_ in the same equation which reveals presence of high electronic density phenyl ring is preferred.

p*K*_i_ = − 0.53MR_omp_ – 0.39π_omp_ – 0.82σ_p_ + 8.23

*n* = 25, *r*^2^ = 0.84, *s* = 0.28, *F* = 37, *p* < 0.0001, *r*^2^_cv_ = 0.76                                                  (12)

The importance of MR and σ_p_ in Equations (11,12) indicates an electronic transfer reaction between the substituted 3-phenyl ring and some site on the receptor leading to involvement of dispersive forces. This study supports our results of CMR being an important parameter in most of our equations.

It is assumed that at the inversion point the structure of a receptor is forced to change into a new shape, which results in an altogether different type of interaction. A possibility could be that there is more than one binding site, but then it shouldn’t have been binding with the same parameters defined in the first half of the Equation [31]. The only way to confirm this allosteric interaction is by doing crystallographic studies of molecules present on both sides of inversion point.

Some outlier(s) were identified while generating equations, which were not considered in deriving the equation because of their arbitrary behaviour. In Equations (6 and 8), compounds 6 and 3 were treated as outliers, as their CMR values were too low than other derivatives in the series. The reason for anomalous behaviour of other outliers could not be attributed to a specific reason. Outlier identification in all equations was also judged by applicability domain estimation through William’s plot (HAT matrix leverage *vs.*. L-O-O residuals). 

A good range of data points was present on both sides of the parabola (positive and negative) and 95% confidence was present on the inversion point. These two checks confirm the quality of our conclusions. 

We came across some studies involving 3D-CoMFA (Comparative Molecular Field analysis) methodology [32,33,34,35,36]. Although quite routinely reported; observations from such studies are difficult to compare with already established QSAR studies. CoMFA with its current status is semiquantitative in nature and does not qualify for quantitative SAR. Results generally are 3D pictures, which cannot be compared precisely. Moreover, the terms used to generate a regression-based model are based on principle components. These terms will have different composition from dataset to dataset so that comparison is impossible leaving pictures as the only tool to compare and conclude [15,18,22,31]. 

**Figure 1:**
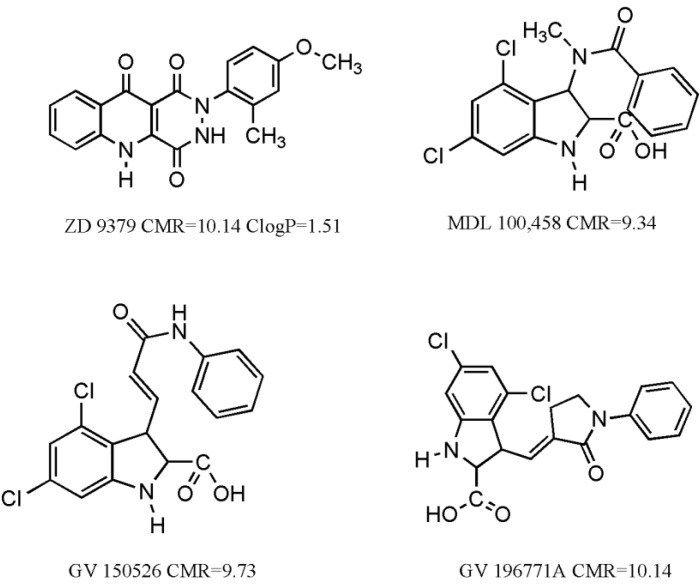
Some molecules having undergone clinical trials for treatment of stroke and/or neuropathic pain.

We have also considered some molecules, which have undergone clinical trials [14] (Figure 1). Interestingly, their CMR values are close to CMR_o_ (10 ± 10%). Such results indicate the quality of experimental work and validate the authenticity of equations. Clog*P* value was calculated for unionic ZD 9379 at pH = 7, which is in alignment with results from Equation (2) that lipophilicity of molecules should be above 0.96 for good activity. The rest have an ionizable carboxylic moiety in their structure and therefore would ionize at physiological pH = 7.4. Clog*P* values would therefore be different at two pH values. In such cases it is better to calculate log*D* values, if necessary. 

Allosteric interaction has been inferred in previous studies by Bender *et al.* [37] and Hansch *et al*. [38]. Their results yield a normal and inverse parabolic relationship with molecular volume and CMR, respectively. It is unknown where our results fit in the allosteric models first proposed by Monod, Wyman and Changeux [39] and later reviewed by Changeux and Edelstein [40]. Koshland *et al.* also proposed their pioneering work on protein allosterism [41]. These models depict changes in protein/receptor containing subunits. In drug discovery, allosteric interaction could be very advantageous in designing newer more efficacious molecules [42].

## 4. Conclusions

The purpose of the study was to conduct comparative a QSAR study on various sets of selective glycine/NMDA antagonists to understand the ligand-receptor interaction mechanism and laterally validate the observations. A multiple linear regression of Hansch type analysis was employed. A test set of 25% molecules was excluded from the training set and their activities were predicted from the modified equations to assess the correlation between internal and external predictivities. CMR was found to be an important parameter in contributing to variation in biological activity. The presence of CMR in the equations reveals that dispersive forces are involved in drug-receptor interaction. Equations (4,8,10) and (6,7) indicated presence of normal and inverse allosterism at glycine site, respectively. Equations (2,3) indicated the importance of hydrophobic interactions. Verloop’s sterimol L1 was also found to be of use in Equation (4). Equations (4,8,10) revealed optimum CMR of 10 ± 10% is required for good activity. Through *I*_a_, Equations (7,8,9,10) show the presence of anionic functionality at 4-position of quinoline/quinolone ring system is not absolutely required for effective binding. All the equations have been laterally validated and compared with previous observations in order to authenticate their results. We believe our equations could be helpful in designing more potent analogs.
